# Fas-L promotes the stem cell potency of adipose-derived mesenchymal cells

**DOI:** 10.1038/s41419-018-0702-y

**Published:** 2018-06-11

**Authors:** Inna Solodeev, Benjamin Meilik, Ilan Volovitz, Meirav Sela, Sharon Manheim, Shai Yarkoni, Dov Zipori, Eyal Gur, Nir Shani

**Affiliations:** 1 The Department of Plastic and Reconstructive Surgery, Tel Aviv Sourasky Medical Center, Sackler Faculty of Medicine, Tel Aviv University, Tel Aviv, Israel; 2 The Cancer Immunotherapy lab, The Neurosurgery department, Tel Aviv Sourasky Medical Center, Sackler Faculty of Medicine, Tel Aviv University, Tel Aviv, Israel; 3 Cellect Biotherapeutics Ltd, 23 Hata’as St., Kfar Saba, Israel; 4 The Department of Molecular Cell Biology, Weizmann Institute of Science, Rehovot, Israel

## Abstract

Fas-L is a TNF family member known to trigger cell death. It has recently become evident that Fas-L can transduce also non-apoptotic signals. Mesenchymal stem cells (MSCs) are multipotent cells that are derived from various adult tissues. Although MSCs from different tissues display common properties they also display tissue-specific characteristics. Previous works have demonstrated massive apoptosis following Fas-L treatment of bone marrow-derived MSCs both in vitro and following their administration in vivo. We therefore set to examine Fas-L-induced responses in adipose-derived stem cells (ASCs). Human ASCs were isolated from lipoaspirates and their reactivity to Fas-L treatment was examined. ASCs responded to Fas-L by simultaneous apoptosis and proliferation, which yielded a net doubling of cell quantities and a phenotypic shift, including reduced expression of CD105 and increased expression of CD73, in association with increased bone differentiation potential. Treatment of freshly isolated ASCs led to an increase in large colony forming unit fibroblasts, likely produced by early stem cell progenitor cells. Fas-L-induced apoptosis and proliferation signaling were found to be independent as caspase inhibition attenuated Fas-L-induced apoptosis without impacting proliferation, whereas inhibition of PI3K and MEK, but not of JNK, attenuated Fas-L-dependent proliferation, but not apoptosis. Thus, Fas-L signaling in ASCs leads to their expansion and phenotypic shift toward a more potent stem cell state. We speculate that these reactions ensure the survival of ASC progenitor cells encountering Fas-L-enriched environments during tissue damage and inflammation and may also enhance ASC survival following their administration in vivo.

## Introduction

Fas ligand (Fas-L), a member of the tumor necrosis factor (TNF) family, is a type II transmembrane protein expressed on the surface of immune cells, such as lymphocytes, natural killer (NK) cells, and macrophages. Upon binding to its receptor (CD95 or Fas receptor (FasR)^1^, the intracellular ‘death-inducing signaling complex’^2^, which includes the aspartate-specific cysteine protease, caspase-8^3–5^, its adaptor/activator, FADD,^6,7^ and its modulator, c-FLIP (FLICE (i.e., caspase-8) inhibitory protein) forms^8^. Caspase-8 is then activated and undergoes proteolytic processing, allowing it to leave the complex^9^, activate caspase-3/7 and induce apoptosis. Fas-L has been implicated in immune system homeostasis maintenance and has been suggested a guardian against autoimmunity^1^. Yet, increasing evidence demonstrate involvement of Fas-L signaling in onset of additional cellular responses, such as inflammation^10–12^, proliferation^13–16^ regeneration^17^, and cancer progression^18^. The relatively ubiquitous expression of CD95 (FasR) in a variety of cells and tissues, corroborates this new line of thought.

Mesenchymal stem cells (MSCs) are multipotent cells that can be produced from most adult body tissues. MSCs are characterized by their adherence to plastic, their ability to differentiate, in culture, to bone, fat and cartilage, and their expression of a set of distinct surface markers^19^. Use of MSCs for various regenerative and immunosuppressive clinical indications has been suggested and their efficacy is currently being evaluated in numerous clinical trials. Despite their considerable potential, transition of MSC use into routine clinical practice has not been achieved to date, partly due to their poor long-term survival following administration^20–22^. As MSCs are known to migrate to inflamed and damaged regions and as they are known to express CD95, it was previously suggested that Fas-L-induced apoptosis may play a major role in their rapid death in vivo^23^. This notion is further supported by the high Fas-L-dependent apoptosis rates observed in cultured bone marrow (BM)^16,24–26^ and fetal blood^27^ MSCs. Importantly, however, Fas-L response in BM-MSCs may have a dual effect as low concentrations of Fas-L were reported to induce only ERK-1/2-dependent proliferation of BM-MSCs, whereas higher doses induced apoptosis and inhibited differentiation^16^.

Adipose-derived stem cells (ASCs), first described in 2002^28^, are MSCs derived from the stromal vascular fraction (SVF) of subcutaneous fat. Although they have been shown to share all characteristics and most of the regenerative and immunosuppressive properties described for bone marrow (BM) MSCs^29^, they also display tissue-specific characteristics^30–32^. Freshly isolated SVF has recently been suggested as an alternative to cultured ASCs to be used within the surgical arena, immediately following their harvest, for regenerative and immunosuppressive purposes. Importantly, the use of freshly isolated autologous SVF for cellular therapies will significantly decrease the cost and time of treatment and regulatory burden compared with cultured ASCs^33,34^. The therapeutic efficacy of SVF transplantation has been established in various pre-clinical disease models and its use for various clinical indications is currently being evaluated in clinical trials^34^.

Given the reported correlation between MSC response to Fas-L death signaling and their survival in vivo^23^, we aimed to evaluate ASC response to Fas-L. ASC responses to Fas-L treatment were characterized by a simultaneous increase in PI3K- and MEK-dependent proliferation and caspase-dependent apoptosis that resulted in doubling of the ASC counts. In addition, phenotypic changes were noted, manifested by altered CD105 and CD73 surface marker levels, increased large colony forming unit-fibroblast (CFU-F) production and increased bone differentiation potential. Overall, Fas-L treatment of either human SVF (Passage 0) or later passage ASCs did not result in their eradication, but, rather, in increased cell yields and phenotypic changes.

## Results

### Simultaneous cell proliferation and apoptosis following Fas-L treatment of SVF cells (Passage 0 (P0) ASCs)

**Fig. 1:**
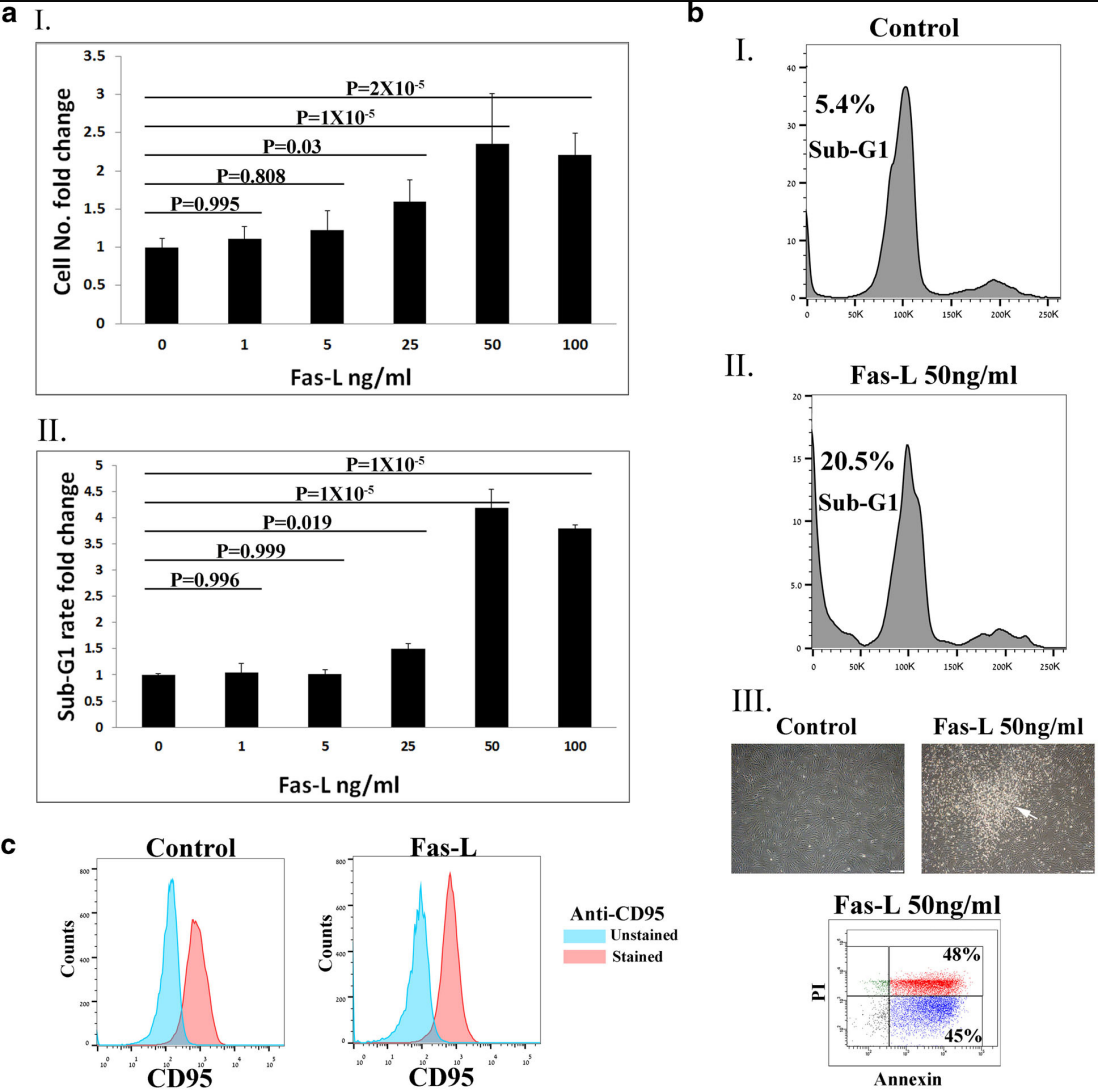
Fas-L treatment promotes both apoptosis and proliferation of SVF (P0 ASCs) in culture. Human SVF cells at P0 were cultured for 14 days, under normal culture conditions or with increasing concentrations of Fas-L. Cell numbers **a**I and sub-G1 proportions **a**II and **b**I and II were calculated by standard cell counts and by propidium iodide (PI) cell cycle flow cytometry analysis, respectively. The experiment was repeated three times using cells from three independent patients. Data are presented as the mean ± standard deviation. **b**III Images of cultured P0 ASCs cells with or without 50 ng/ml Fas-L. Floating cells of treated cells (marked by an arrow) were collected and examined using the annexin PI assay. All floating cells were determined to be in different apoptosis stages. **c** CD95 (Fas) expression on Fas-L treated and untreated cells was examined by Flow cytometry analysis. CD95 demonstrated similar expression levels in Fas-L treated (50 ng/ml) and untreated cells.

To evaluate the effect of Fas-L treatment on SVF cells, we compared the cell yield and apoptosis rate of P0 ASCs cultured for 14 days immediately following their isolation from fat in normal growth medium in the presence vs. absence of increasing Fas-L concentrations. As shown in Fig. 1aI, II, no response to Fas-L concentrations of up to 25 ng/ml was recorded. In contrast, at concentrations of 25 ng/ml and above, P0 ASCs demonstrated a dose-dependent increase in apoptosis rates (Fig. 1aII, b). Nonetheless, the cell counts were double or more (Fig. 1aI). A ~doubled cell yield following 50 ng/ml Fas-L treatment was demonstrated in P0 ASCs extracted from 10 independent patients (Supp. Table 1). Fas-L response was most probably transduced through the canonical FasR (CD95) as ~ 80% of ASCs, independent of Fas-L treatment, expressed CD95 (Fig. 1c). Thus, ≥25 ng/ml Fas-L induces simultaneous proliferation and apoptosis of SVF cells.

### Fas-L treatment of SVF cells (P0 ASCs) leads to an increase in the CD105-low and CD73-high cell populations of passage 0 and 1 ASCs

**Fig. 2:**
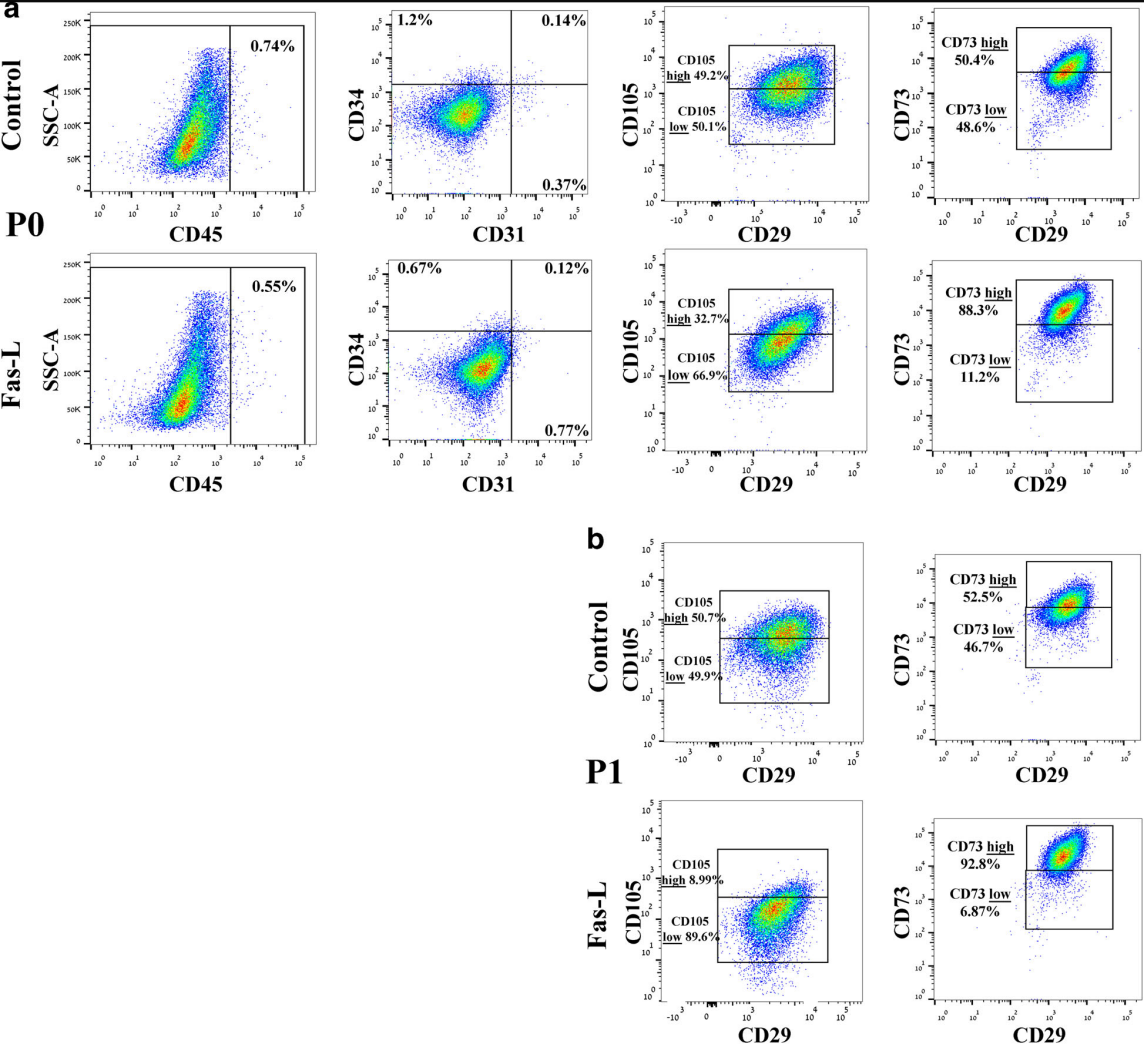
Fas-L treatment leads to an increase in CD105-low and CD73-high subpopulations of cultured SVF cells at passages 0 and 1. P0 (**a**) and P1 ASCs (**b**), cultured under normal culture conditions or with 50 ng/ml Fas-L, were examined for their surface marker expression of CD45, CD31, CD34, CD29, CD105, and CD73 by flow cytometry. The experiment was repeated two times using cells from two independent patients giving the same trend

Next, we examined whether Fas-L-dependent apoptosis and proliferation of P0 ASCs also resulted in phenotypic changes. To this end, we compared the surface marker profile of Fas-L-treated P0 ASCs at passage 0 and 1 to that of untreated controls, using our 7-color flow cytometry panel. As expected, at passage 0, fewer than 2% of the untreated cells expressed CD45, CD31, and CD34, close to a 100% expressed MSC cell markers CD29 and CD73 and CD105 (Fig. 2a). A similar expression profile was recorded at passage 1 in untreated cells (Fig. 2b and data not shown). Despite a significant similarity in the surface marker expression pattern of untreated and Fas-L-treated passage 0 cells, a higher percentage of CD105-positive cells expressing low levels of CD105 was noted in Fas-L-treated cells as compared with untreated control cells (CD105-low) (66.9% compared with 50.1%, respectively). In addition, Fas-L-treated passage 0 cells displayed a higher percentage of CD73-positive cells expressing high CD73 levels (CD73 high) (88.3% compared with 50.4%), as compared with untreated control cells (Fig. 2a). A further increase in the relative percentages of these two subpopulations was noted in passage 1, with 89.6% of Fas-L-treated CD105-positive cells, demonstrating CD105-low and 92.8% of Fas-L-treated CD73-positive cells demonstrating CD73-high, as compared with only 49.9 and 52.5%, respectively, of the passage 1 untreated control (Fig. 2b). Thus, Fas-L treatment of SVF cells leads to the enrichment of CD105-low and CD73-high cells.

### Fas-L-treated ASCs display similar fat differentiation and higher bone differentiation compared with untreated cells

**Fig. 3:**
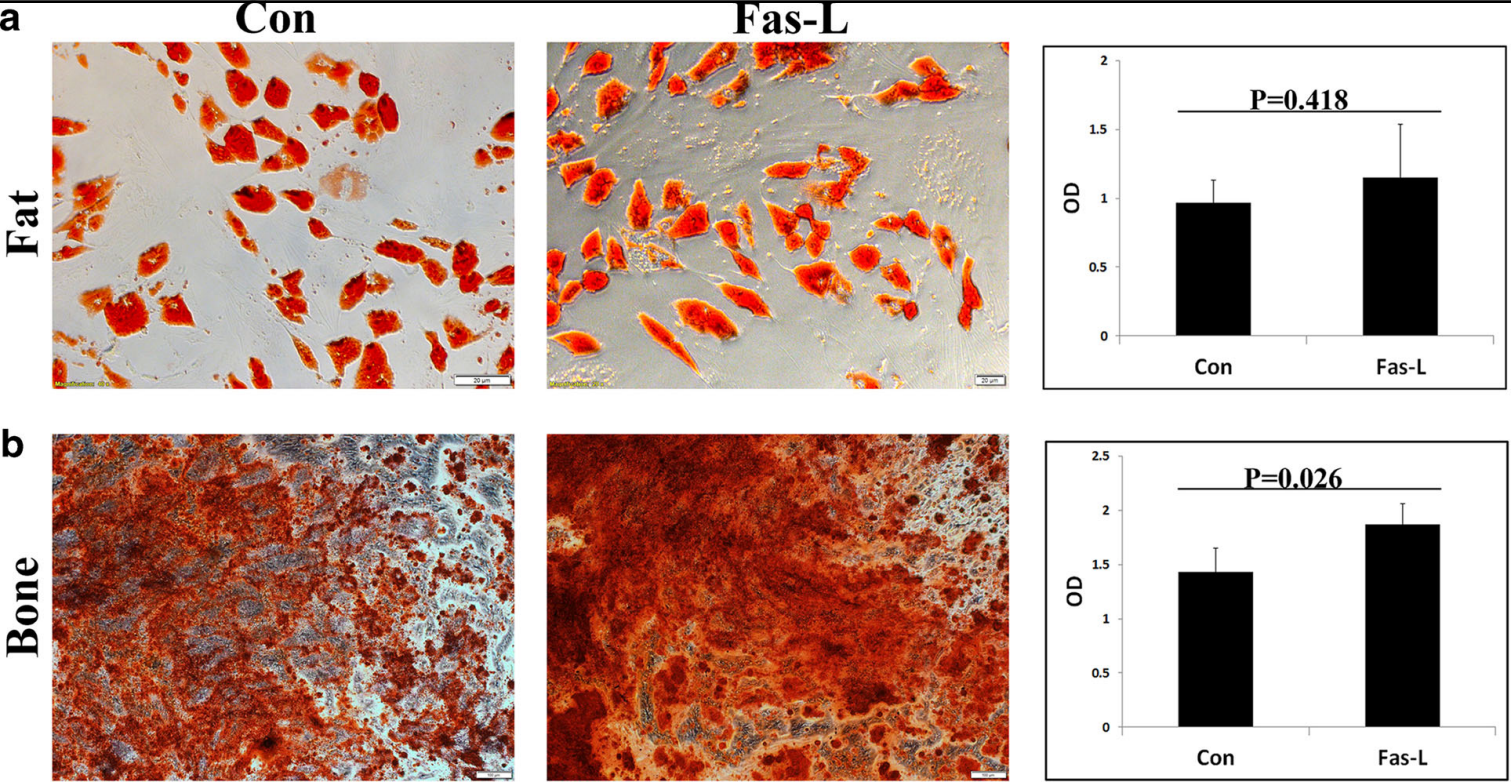
Fas-L treatment increases the bone differentiation capacity of cultured SVF cells (P0 ASCs). Human SVF cells at P0 were cultured for 14 days under normal culture conditions or with 50 ng/ml Fas-L. Treated and untreated cells were than passaged and induced to undergo fat or bone differentiation using designated differentiation media at P1. Differentiation into bone and fat was detected by Alizarin red and Oil red O staining, respectively. Cells differentiated to fat (**a**) and bone (**b**) were then photographed and the stain was extracted and quantified. The experiment was repeated three times using cells from three independent patients giving the same trend

It was previously reported that different ASC subpopulations, characterized by distinct surface marker expression, demonstrate different differentiation potentials^35–38^. Comparison of the differentiation of Fas-L-treated vs. untreated passage 1 ASCs (These ASCs received Fas-L treatment at passage 0) with fat and bone revealed similar fat differentiation in Fas-L-treated and untreated cells, but enhanced bone differentiation following Fas-L treatment (Fig. 3a, b, respectively).

### Fas-L treatment of SVF cells promotes the production of large CFU-Fs

**Fig. 4:**
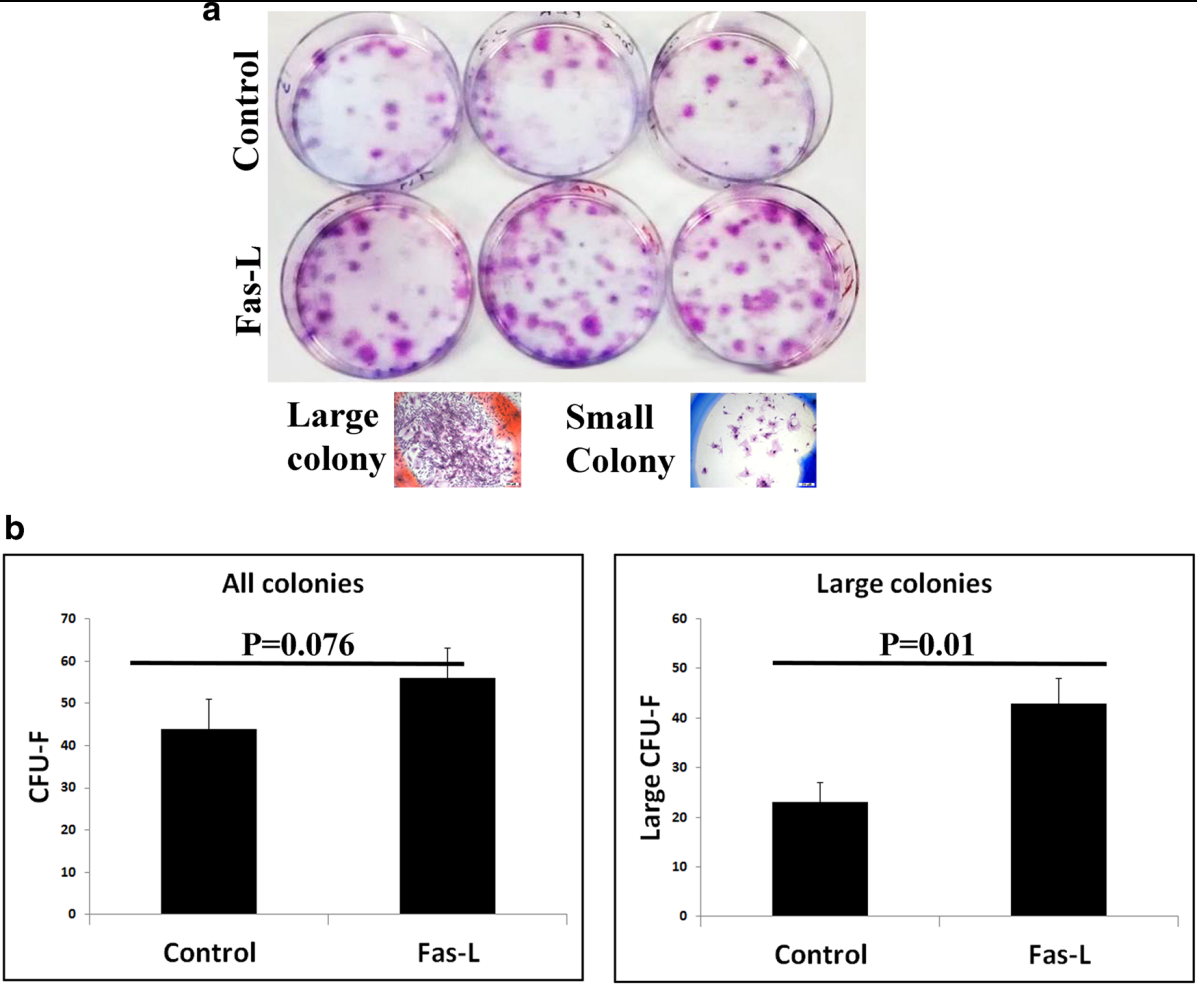
Fas-L treatment of SVF cells increases large CFU-F formation. Human SVF cells were cultured at low densities, for 21 days, under normal culture conditions or with 50 ng/ml Fas-L. Colonies were then stained by Giemsa and counted. Colonies were photographed (**a**) and the total number of colonies and of large colonies (>100 cells) was compared between Fas-L-treated and untreated cells **b**. The experiment was repeated two times using cells from two independent patients giving the same trend

Next, we evaluated the capacity of Fas-L-treated and untreated SVF cells to form CFU-Fs. Although at a first glance, it seemed that Fas-L-treated cells formed significantly more CFU-Fs than untreated cells (Fig. 4a), careful microscopic evaluation revealed that the CFU-Fs population was comprised of both large and small CFU-Fs, where the large colonies were of > 100 cells (Fig. 4a bottom). As can be seen in Fig. 4b, whereas the total number of colonies did not reveal a significant difference between Fas-L-treated and untreated cells, a significantly higher number of large colonies was found in Fas-L-treated SVF cells compared with untreated cells.

### Fas-L treatment of passage 3 ASCs results in a phenotype shift similar to that observed in SVF cells (P0 ASCs)

**Fig. 5:**
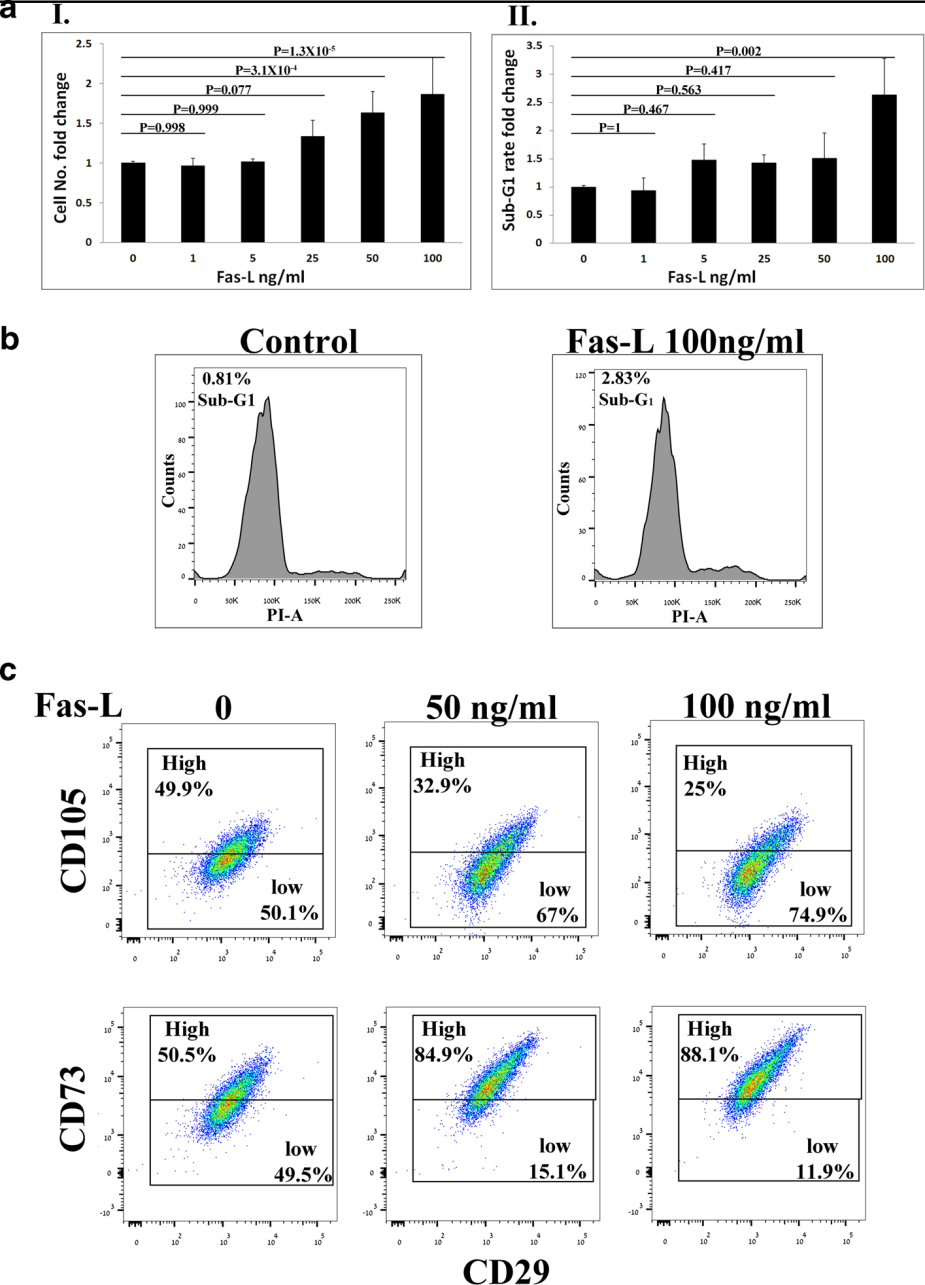
Fas-L-treated P3 ASCs and Fas-L-treated SVF cells (P0 ASCs) display a similar phenotype. ASCs (P3) were cultured for 6 days under normal culture conditions or with increasing Fas-L concentrations. Cell numbers (**a**I) and sub-G1 proportions **a**II and **b** were examined by standard cell counts and by propidium iodide (PI) cell cycle flow cytometry analysis, respectively. The experiment was repeated three times using cells from three independent patients. Data are presented as the mean ± standard deviation. P3 ASCs cultured under normal culture conditions or with 50 ng/ml Fas-L, surface marker expression of CD29, CD105, and CD73 was examined by flow cytometry (**c**)

All the experiments thus far evaluated the effect of Fas-L treatment on SVF cells (P0 ASCs), immediately following their isolation from fat. In order to examine whether Fas-L can induce a similar effect when added to passage 3 (P3) ASCs that were expanded in culture for a longer period of time, P3 ASCs were cultured under normal conditions or in the presence of increasing concentrations of Fas-L. As with P0 ASCs, Fas-L treatment led to a concentration-dependent increase in proliferation (Fig. 5aI) and to an increase in the CD105-low and CD73-high populations (Fig. 5c) of P3 ASCs. In contrast, P3 ASCs seemed more resistant to Fas-L apoptotic signal than P0 ASCs and displayed a significant increase in apoptosis rates only when a concentration of 100 ng/ml was used (Fig. 5aII, b).

### Caspase inhibition attenuates Fas-L-dependent apoptosis but not proliferation

**Fig. 6:**
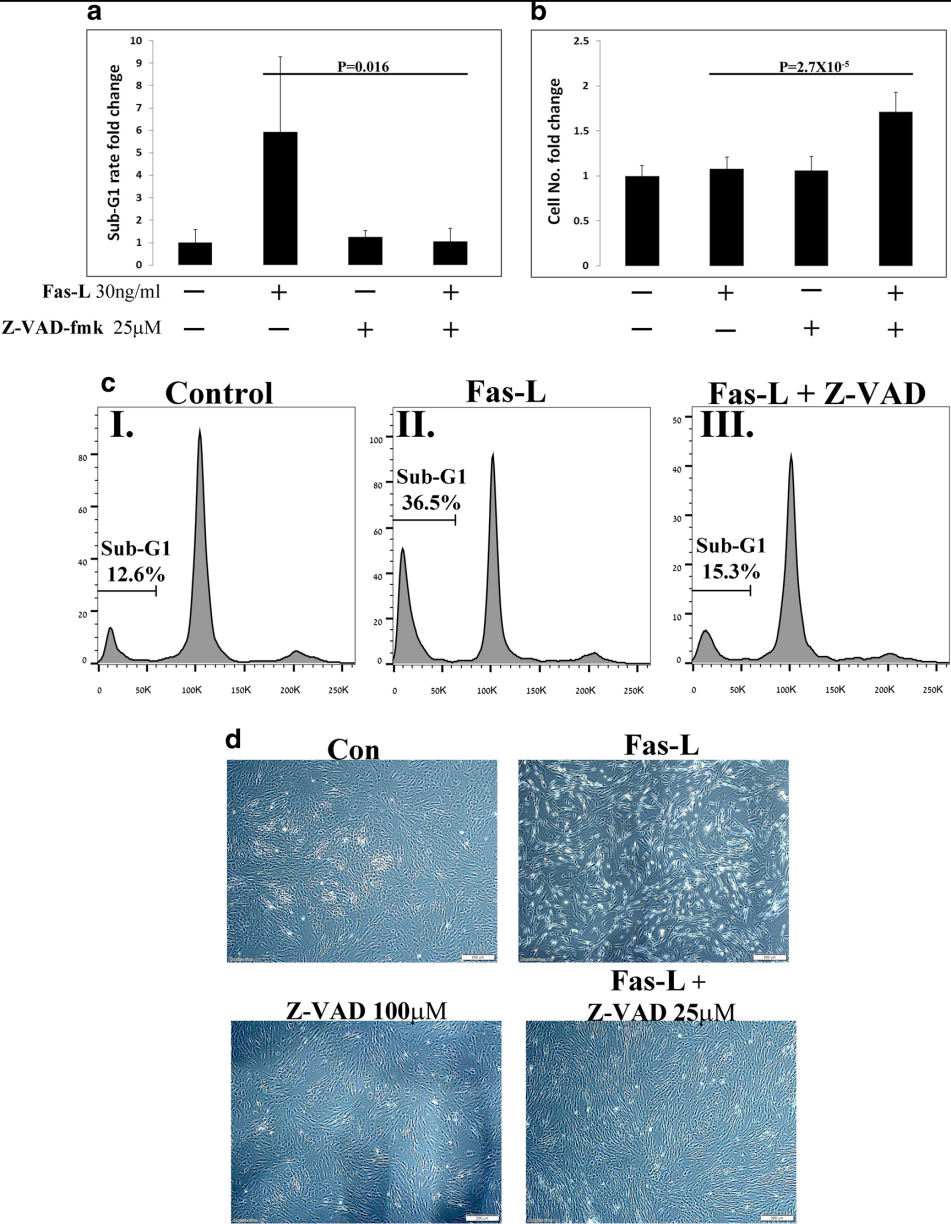
Caspase inhibition prevents Fas-L-induced apoptosis but not Fas-L-induced proliferation of ASCs. ASCs (P2-3) were treated for 4 days with the Fas-L, caspase inhibitor Z-VAD-fmk or both. Sub-G1 proportions **a** and **c** and cell numbers (**b**) were determined by PI cell cycle flow cytometry analysis and standard cell counts, respectively. The experiment was repeated three times using cells from three independent patients. Data are presented as the mean ± standard deviation. **d** Representative images of ASCs under the different conditions are shown

The dual effect of Fas-L on the apoptosis and proliferation of ASCs, can be mediated solely by canonical Fas-L signaling or through two distinct pathways. To distinguish between these possibilities, ASCs were either left untreated, treated with Fas-L only or treated simultaneously with Fas-L and with the pan-caspase inhibitor Z-VAD-fmk, for 4 days. Importantly, as P3 ASCs are more resistant to Fas-L induced apoptosis (See Fig. 5a, b), a different Fas-L derivative (mega-Fas-L), which efficiently induces simultaneous apoptosis (Fig. 6a) and proliferation in P2-3 ASCs (Supp. Table 2), was used in the current experiments. As expected, Fas-L treatment led to a sharp increase in ASC apoptosis, as demonstrated by an increase in the sub-G1 population of treated cells (Fig. 6a–c). Although apoptosis was considerably attenuated by the addition of Z-VAD-fmk (Fig. 6a, c and d), Fas-L-induced ASC proliferation was not affected, and the cell yield ~doubled over the 4-day treatment period when a combination of Fas-L and Z-VAD-fmk was used (Fig. 6b and Supp. Table 3). The increased expansion of cells treated with the combination of Fas-L and Z-VAD-fmk was due only to reduced Fas-L-induced apoptosis through caspase inhibition, as no such increased expansion was observed in cells treated with Z-VAD-fmk only. In the current experiments treatment of Fas-L only did not demonstrate an increased cell yield compared with untreated cells (Fig. 6b and Supplementary Table 3). This was due to the shorter duration of this experiment (4 days) as compared with previous experiments (7–14 days) and not to the change in the Fas-L derivative as treatment with mega-Fas-L for 7 days led to a ~doubled cell yield (Supplementary Table 2).

### Inhibition of PI3K and MAP kinases ERK-1/2 but not of JNK suppresses Fas-L-induced proliferation of ASCs

**Fig. 7:**
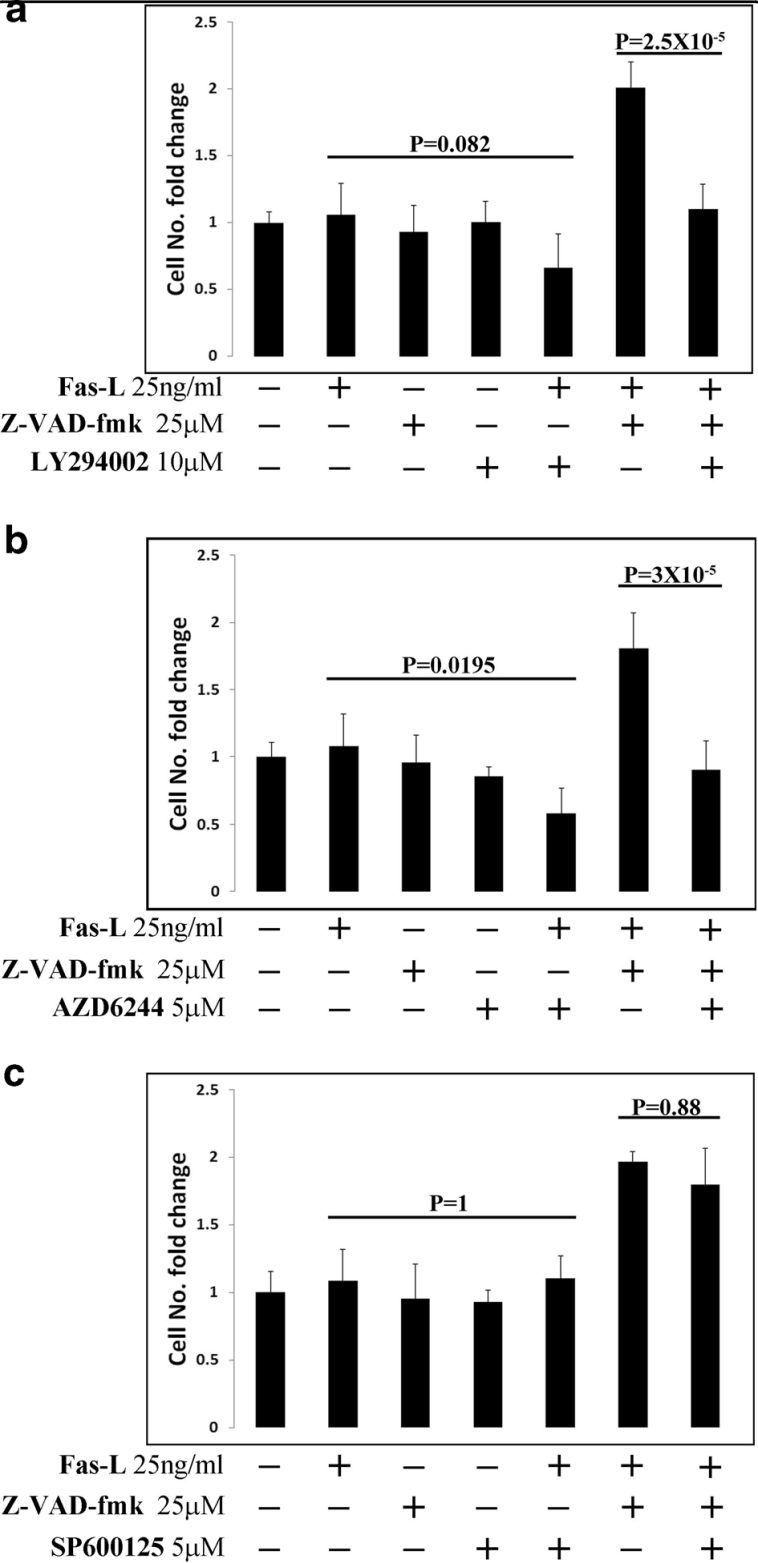
Inhibition of PI3K/Akt and MAP kinases ERK-1/2 but not JNK attenuates Fas-L-induced proliferation. Cultured ASCs (P2-4) were treated with the indicated combination of mega-Fas-L, Z-VAD-fmk, LY294002 (PI3K inhibitor), AZD6244 (ERK-1/2 inhibitor), and SP600125 (JNK inhibitor). Four days later, cells were removed from plates by trypsin and cells were counted using a standard cell counter. Passage 2–4 ASCs were used in all experiments. The experiment was repeated three times using cells from three independent patients. Data are presented as the mean ± standard deviation

To identify the intracellular pathways that control Fas-L-induced proliferation in ASCs, we independently inhibited PI3K/Akt, ERK-1/2, and JNK signaling pathways, all of which were previously shown to be activated by Fas-L^16,39–43^. Inhibitor concentrations were calibrated to the maximal doses, which did not interfere with basal ASC proliferation (Fig. 7a–c and data not shown). After 4 days in culture with both Fas-L and either PI3K (Fig. 7a) or ERK-1/2 (Fig. 7b) inhibitors, reduced ASC counts were measured as compared with cells treated with Fas-L only (Fig. 7a, b, respectively), indicating inhibition of the pro-proliferative but not of the pro-apoptotic effect of Fas-L by the inhibitors. These findings were confirmed upon culture of ASCs with Fas-L, Z-VAD-fmk, and either PI3K (Fig. 7a) or ERK-1/2 (Fig. 7b) inhibitors, which yielded cell quantities comparable to untreated control, whereas a ~doubled cell yield was demonstrated following treatment with Fas-L and Z-VAD-fmk (Fig. 7a, b). In contrast, JNK inhibition concomitant to Fas-L stimulation, yielded cell numbers similar to those obtained following Fas-L treatment only (Fig. 7c). Similarly, the simultaneous addition of Fas-L, Z-VAD-fmk, and a JNK inhibitor resulted in population doubling as obtained upon treatment with Fas-L and Z-VAD (Fig. 7c).

## Discussion

Fas-L has long been considered a death ligand that promotes apoptosis by binding to its receptor CD95 (FAS) and activating caspases. However, accumulating data now suggest that Fas can transmit various non-apoptotic signals that may have far-reaching implications on a range of physiological and disease conditions^44,45^. Surprisingly, we found that Fas-L signaling simultaneously activates apoptosis and proliferation of both freshly derived and cultured ASCs. Despite significant apoptosis induction, Fas-L signaling led to increased cell expansion (~doubled cell quantities) of ASCs and to a phenotypic shift, evidenced by a change in MSC-specific marker expression, ability to produce higher quantities of early CFU-Fs, and increased bone differentiation potential. Further assessments demonstrated that the apoptotic and proliferative Fas-L cues were not interconnected and that blocking the apoptotic signal had no effect on the proliferative signal and vice versa. In agreement with previous reports, proliferation activation was attenuated by both PI3K and MEK inhibitors, indicating the involvement of these pathways in the transmission of the Fas-L signal. In light of the increasing application of both SVF cells and ASCs for regenerative purposes, we envision the use of Fas-L activation as an efficient tool to increase cell yields and to possibly also augment cell phenotypes for specific clinical indications, such as bone regeneration. Given the apoptotic response and reduced cell yield previously reported following Fas-L treatment of BM-MSCs^16,24–26,46^, the ability of ASCs to respond to Fas-L signaling by a proliferative burst may serve as a clinical advantage upon their delivery, particularly when exposed to Fas-L-presenting immune cells commonly enriched in inflamed areas.

Several studies exploring Fas-L signaling have found non-apoptotic Fas-L signaling to be distinct from its apoptotic signaling. Moreover, these works noted that cells undergoing non-apoptotic Fas-L signaling often do not respond to apoptosis-related Fas-L signaling either due to their normal tissue tendency or in apoptosis-resistant tumor cells bearing a defective Fas-L signaling pathway^39,41,42,47,48^. However, a recent report found Fas-L to simultaneously induce apoptosis and pro-inflammatory signaling in various cells, suggesting the induction of an immunological response by dying cells^10^. In contrast to this report, in which the outcome of Fas signaling was cell death, we demonstrate an overall increase in SVF and ASC counts upon Fas-L signaling, due to simultaneous apoptosis and proliferation activities. The increased final yield of ASCs following Fas-L treatment, is probably due to the fact the more cells undergo proliferation than apoptosis in response to Fas-L treatment, tipping the balance toward increased expansion. This phenomenon may be partly explained by our observation that Fas-L induced apoptosis is rapid and occurs mostly on the first 24 h following its addition (data not shown), whereas Fas-L-induced proliferation may continue for longer periods. This is partly evident by the observation that Fas-L treatment of ASCs for 4 days resulted in expansion similar to untreated control, whereas 6 days treatment resulted in ~doubled ASC expansion. The delicate balance between apoptosis and proliferation following Fas-L treatment will be further studied in the future. We speculate that Fas-L induces proliferation of ASCs and possibly also of their stem progenitor cells within fat tissue, allow them to respond to tissue damage and the consequent infiltration of Fas-L-expressing immune cells, promoting their survival in order to take part in tissue repair and regeneration. ASC response to Fas signaling is different from that of BM-MSCs, which have been reported to respond to Fas signaling by a reduction in cell numbers, owing to apoptosis^16,24–26,46^. Moreover, a study which used the same Fas-L derivative as the one used in the current study (super-Fas-L), reported a reduction of BM-MSCs proliferation in response to Fas-L at concentrations as low as 5 ng/ml Fas-L, whereas we measured increased ASC expansion at concentrations as high as 200 ng/ml (data not shown)^16^. Interestingly, although 25 ng/ml Fas-L were reported to induce increased apoptosis of BM-MSCs, a milder dose of 0.5 ng/ml reportedly induced only ERK-1/2-dependent proliferation of BM-MSC^16^. In primary foreskin fibroblasts, cells expressing low CD95 levels were previously shown to undergo proliferation following Fas-L binding, whereas overexpression of exogenous CD95, in the same cells, led to apoptotic induction by Fas-L binding^15^. It is thus possible that all MSCs can respond to Fas signaling by proliferation, apoptosis or both, depending on the Fas-L concentrations and on their specific sensitivity to Fas-L signaling.

Fas signaling in ASCs also resulted in a phenotypic change demonstrated by an increase in the percent of cells with low surface expression of CD105 (CD105-low) and high surface expression of CD73 (CD73-high). CD105 and CD73 are both well-established MSC markers and have also been specifically defined as ASC markers^19,49^. In accordance with previous reports, which demonstrated improved bone differentiation of CD105-low compared CD105-high MSCs^35,38^, Fas-L treatment led to both an increase in CD105-low ASCs and to their improved bone differentiation potential compared with untreated control. An additional report demonstrated increased osteogenic potential following overexpression of CD73 in MC3T3-E1 preosteoblasts, establishing the importance of CD73 expression to bone differentiation^50^. These findings again are in accordance with the presented increased proportion of CD73-high ASCs following Fas-L treatment, in correlation with the improved bone differentiation potential. ASCs have the capacity to differentiate to both fat and bone, however, because they originate from fat tissue, they show an increased tendency toward fat differentiation (data not shown). We therefore speculate that the increased bone differentiation capacity of Fas-L-treated ASCs may indicate increased stemness compared with untreated controls. The larger CFU-Fs observed following Fas-L treatment of freshly isolated SVF cells, which have previously been associated with more primitive stem cell progenitor cells^51^, partly supports this line of thought.

In order to elucidate the signaling pathways that promote Fas-L-induced apoptosis and/or proliferation, we utilized inhibitors of various pathways that were previously demonstrated to promote Fas-controlled apoptotic and non-apoptotic signaling. It is established that the Fas-dependent apoptotic signal is mediated by caspase activation^18^. Importantly, we found that inhibition of caspases by the pan-caspase inhibitor Z-VAD-fmk led to near-complete inhibition of apoptosis in Fas-L-treated ASCs but had a minor to negligible effect on proliferation. This strongly suggests that unlike previous reports, which demonstrated induction of non-apoptotic pathways by caspase-8^12,39,42,52^, non-apoptotic signaling triggered by Fas-L in ASCs is caspase-independent. Fas-L-dependent non-apoptotic functions were previously demonstrated to depend on JNK^42^, ERK^16,39,40^, or PI3K^41,43^. We found that Fas-L-dependent proliferation of ASCs could be inhibited by ERK and PI3K inhibitors but not by a JNK inhibitor. Importantly, none of the inhibitors significantly abrogated Fas-L-dependent apoptosis in ASCs. Thus, although Fas-L simultaneously induces apoptosis and proliferation of ASCs, the signaling that promotes each functional outcome seems to divert downstream to the Fas receptor. Further work will be necessary to elucidate the exact mechanisms by which each signal is transferred on the molecular level.

The current report demonstrates, for the first time, that Fas-L signaling simultaneously promotes apoptosis and proliferation of freshly isolated and cultured human ASCs, leading to an overall increase in cell expansion and to a phenotypic change. We hypothesize that these observations represent a physiological response of ASC progenitor cells to Fas-L-enriched conditions typical to damaged or inflamed tissue. Overall, cell proliferation and phenotypic adaptation are promoted, allowing for efficient tissue regeneration and repair. In the context of regenerative medicine, the ability to survive and proliferate under Fas-L-enriched conditions may assist in the survival of SVF cells and ASCs following their clinical administration in vivo.

## Materials and methods

### Materials

Recombinant human super-Fas-L was obtained from Enzo Life Sciences (L¨orrach, Germany) and recombinant human mega-Fas-L from AdipoGen (San Diego, CA, USA). z-VAD.fmk (pan-caspase inhibitor), LY294002 (PI3K inhibitor), and SP600125 (JNK inhibitor) were obtained from Merck Biosciences (Darmstadt, Germany) and AZD6244 (ERK-1/2 inhibitor) was obtained from Cayman Chemical (Michigan, USA).

The following antibodies were used for flow cytometry stainings: anti-human CD95 (APO-1/Fas) APC and APC mouse IgG1 k isotype control (eBioscience San Diego, CA), anti-human CD31 APC and mouse IgG1 isotype control APC, anti-human CD34 PE, and mouse IgG1 k Isotype control PE (PeproThec London, UK), anti-human CD29 Alexa Fluor 488 and Alexa Fluor 488 mouse IgG1 k isotype control, anti-human CD105 PerCP/Cy5.5 and PerCP/Cy5.5 mouse IgG1 k Isotype control, anti-human 73 PE/Cy7 and PE/Cy7 mouse IgG1 k isotype control (BioLegend (San Diego, CA), anti-human CD45 BD Horizon BV650 and BV650 mouse IgG1, k isotype control BD Biosciences (San Jose, CA).

### Human primary SVF cell isolation

Subcutaneous adipose tissue samples were obtained from patients undergoing plastic surgery. All procedures were performed in accordance with the Declaration of Helsinki and approved by the ethics committee of Tel Aviv Sourasky Medical Center. Written, informed consent was obtained from all patients in advance. All samples were waste materials collected as a byproduct of surgery. The mean age of the patients was 46.1 + −11.7 years, the mean BMI was 29.3 + −4.8 kg/m^2^. SVF cells were isolated from the subcutaneous human lipoaspirates using 0.1% collagenase (Sigma, St. Louis, MO, USA), and separated from the fat by centrifugation (15 min, 400 g).

### Cell culture

Human primary SVF cells from adipose tissue were maintained in their undifferentiated state in high-glucose Dulbecco’s modified Eagle’s medium (DMEM) (Gibco, Paisley, Scotland, UK), supplemented with 10% fetal calf serum (Thermo Scientific HyClone, Tauranaga, New Zealand), 60 μg/ml penicillin, 100 μg/ml streptomycin, 50 μg/ml kanamycin, 1 mM sodium pyruvate, 2 mM L-glutamine and non-essential amino acids, under 10% CO_2_ and atmospheric oxygen conditions. Medium was changed twice a week, and cells were passaged once they reached confluence.

### Cell proliferation

SVF were seeded into six-well plates (150,000 cells/well) and cultured for 14 days in either normal growth medium or in growth medium supplemented with different Fas-L concentrations. Medium was refreshed four times during this 14 days culture.

Mature ASCs (P2-P3 ASCs) were seeded into 6 cm plates (90,000 cells/plate) and allowed to attach and grow for 24 h. Then, medium was changed to fresh normal growth medium or growth medium supplemented with different Fas-L concentrations, and cells were further cultured for 7 days. Medium was refreshed twice during the experiment period.

To test the effects of inhibitors on proliferation of mature ASCs (P2 and P3 ASCs), cells were seeded into 24-well plates (10,000 cells/well) and allowed to attach and grow for 24 h. Then, medium was exchanged for fresh normal growth medium or growth medium supplemented with Fas-L or with Fas-L and one of the inhibitors. Cells were then cultured for 4 days. For all treatments, duplicate wells/plates were prepared. At the end of the incubation period, adherent cells were trypsinized, collected and counted with a TC10 Automated Cell Counter (Bio-rad).

### Flow cytometry

#### Surface marker analysis

For surface marker analysis, cells were harvested and incubated (1 h, in the dark) with a 7-color panel containing anti-CD31, anti-CD34, anti-CD29, anti-CD105, anti-human-73, and anti-CD45 antibodies. To exclude dead cells, the samples were stained with ViViD (violet viability dye, Molecular Probes, Invitrogen, Eugene, OR, USA), according to the manufacturer’s protocol. CD95 staining was performed using a 2-color panel containing anti-CD95 antibody and ViViD. All antibodies were used at the dilution recommended by the manufacturer. Appropriate single-stained and isotype controls samples were prepared and analyzed.

#### Cell cycle analysis

Cells were harvested and fixed with 70% ethanol/phosphate-buffered saline, treated with RNaseA 0.4 mg/ml (Sigma) and stained with propidium iodide (PI) 0.1 mg/ml (Sigma).

#### Annexin/PI analysis

Cells were stained with an Annexin-APC/PI using a dedicated kit (BioLegend). All labeled cells were analyzed using a FACS Canto II flow cytometer (Becton Dickinson, San Jose, CA, USA). At least 10,000 events were recorded. Data analysis was performed using the FlowJo software (Tree Star, Ashland, OR, USA).

### Differentiation

Prior to differentiation, Fas-L-treated and -untreated ASCs were seeded in 24 wells and were cultured until a confluent state was reached. Once seeded in the 24 well both groups of cells were cultured in regular medium without Fas-L. In addition, no Fas-L was added to the cells throughout the differentiation process. Cell counts of previously Fas-L-treated and -untreated cells were performed at the end of the differentiation process.

### Adipogenic differentiation

Confluent cells were cultured in adipogenic medium containing 10 μg/ml insulin, 1 × 10^−6^ M dexamethasone, 0.5 mM 3-isobutyl-1-methylxanthine (IBMX) and 50 μM indomethacin (all from Sigma). The induction medium was replaced every 3–4 days. After 21 days, the cells were fixed with 4% formalin (20 min at room temperature (RT)) and stained with 0.5% Oil Red (Sigma) (10 min, at RT). Following the staining, cells were photographed with an Olympus IX71 microscope (Olympus, Tokyo, Japan), equipped with a DP73 camera. Oil Red O was then extracted with 4% IGEPAL (Sigma) in isopropanol, and then quantified at 520 nm using a TECAN Infinite M200 plate reader (TECAN, Männedorf, Switzerland). Reads were normalized according to cell counts.

### Osteogenic differentiation

Confluent cells were cultured in StemPro osteogenesis differentiation medium (Gibco). The induction medium was replaced every 3–4 days. After 21 days, the cells were fixed with 4% formalin (20 min at RT) and stained with 2% Alizarin red (Sigma), pH 4.2 (10 min, at RT). Photographs were taken using an Olympus IX71 microscope with a DP73 camera. Alizarin red was extracted with extraction solution (0.5 N HCL and 5% sodium dodecyl sulphate) and quantified at 415 nm using a TECAN Infinite M200 plate reader (TECAN, Männedorf, Switzerland). Reads were normalized according to cell counts.

### CFU-F assay

SVF cells (2000) were seeded on a 6-cm dish and cultured for 21 days under normal culture conditions or with medium supplemented with 50 ng/ml Fas-L. Colonies were then fixed with methanol, stained with Giemsa (Sigma) and counted to evaluate CFU-F formation. The total number of colonies and of large colonies (> 100 cells) was quantified (using an Olympus IX71 microscope) and compared between Fas-L-treated and -untreated cells. These experiments were performed in triplicates.

### Statistical evaluation

The statistical analysis was performed using the Minitab 18 software (Minitab Inc., Pennsylvania). The data were subjected to one-way analysis of variance followed by Dunnett’s (for comparison with control group) or Tukey’s multicomparison test. Univariate data were analyzed using paired *t* test. *p* ≤ 0.05 was considered statistically significant.

## Electronic supplementary material

Supplementary table 1

Supplementary table 2

Supplementary Table 3

Supplementary figure legends
