# Supplementary figures and images for: Fas-L promotes the stem cell potency of adipose-derived mesenchymal cells
Source: Cell Death Dis. 2018 Jun 11;9(6):695. doi: 10.1038/s41419-018-0702-y (PMC5995957; doi:10.1038/s41419-018-0702-y)

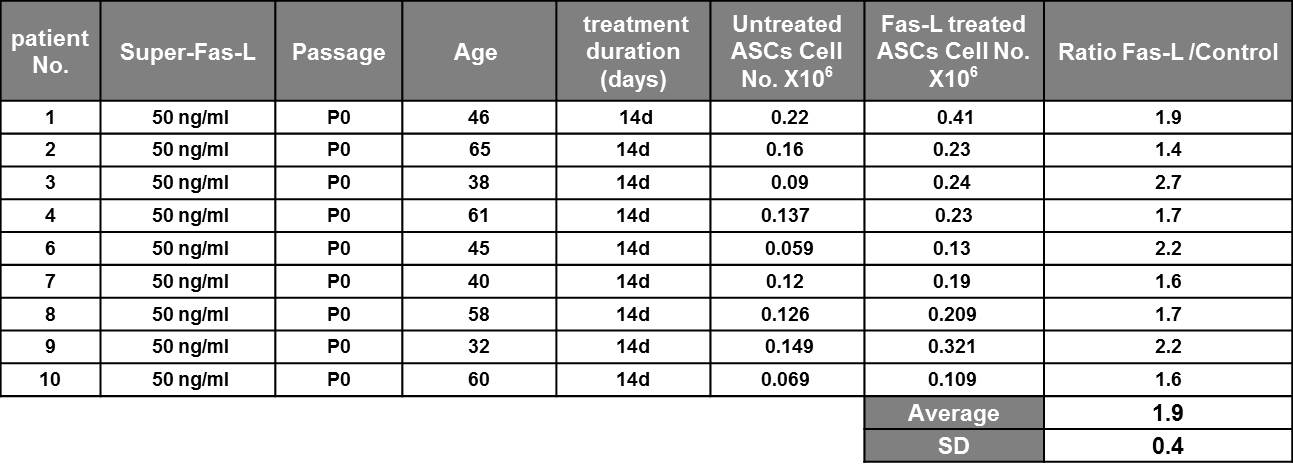

Supplement: Supplementary file 1 — Supplementary table 1 [file 41419_2018_702_MOESM1_ESM.jpg]

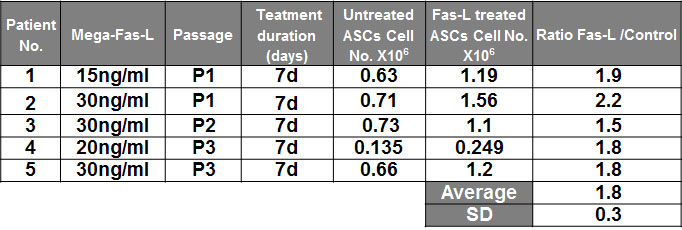

Supplement: Supplementary file 2 — Supplementary table 2 [file 41419_2018_702_MOESM2_ESM.jpg]

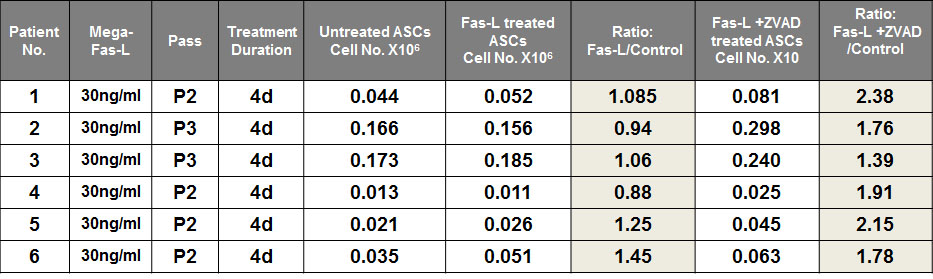

Supplement: Supplementary file 3 — Supplementary Table 3 [file 41419_2018_702_MOESM3_ESM.jpg]
