# Supplementary material for: Fas-L promotes the stem cell potency of adipose-derived mesenchymal cells
Source: Cell Death Dis. 2018 Jun 11;9(6):695. doi: 10.1038/s41419-018-0702-y (PMC5995957; doi:10.1038/s41419-018-0702-y)
Supplement: Supplementary file 4 — Supplementary figure legends [file 41419_2018_702_MOESM4_ESM.docx]

Supplementary table 1. Increased cell yield following Fas-L treatment of SVF cells (P0 ASCs).

Supplementary table 2. Increased cell yield following Mega-Fas-L treatment of Passage 1-3 ASCs for 7 days.

Supplementary Table 3. Increased cell yield following treatment of Passage 2-3 ASCs for 4 days with Mega-Fas-L + ZVAD but not with Mega-Fas-L only.
